# Presentation of an unusual variant of cellular dermatofibroma: a case report

**DOI:** 10.1093/jscr/rjaf257

**Published:** 2025-05-17

**Authors:** Basem H Alshareef, Ghadeer Faiz M Alharthi, Saleha Khan, Shumaila Baig

**Affiliations:** Department of General Surgery, Umm Al-Qura University, Al Taif Road, Makkah 24382, Saudi Arabia; General Surgery Senior Registrar, Al-Noor Specialist Hospital, 3rd Ring Rd, Al Hijrah, Makkah 24241, Saudi Arabia; General Medicine Practice Program, Batterjee Medical College, Prince Abdullah Al-Faisal Street, North, Jeddah 21442, Saudi Arabia; General Medicine Practice Program, Batterjee Medical College, Prince Abdullah Al-Faisal Street, North, Jeddah 21442, Saudi Arabia

**Keywords:** dermatofibroma, histiocytoma, cellular dermatofibroma, case report, soft tissue tumour, neoplasm

## Abstract

Dermatofibromas (DF), also known as cutaneous fibrous histiocytomas, are benign soft-tissue tumours that are typically asymptomatic. These lesions most commonly appear on the distal extremities and are frequently seen in young to middle-aged individuals. Diagnosis is primarily based on histopathological examination, which generally aligns with radiological findings. This report highlights an atypical variant of cellular DF, in a 26-year-old female who presented with a painless foot ulcer on her lower leg. Initially, the lesion was suspected to be a sarcoma. After excision, the diagnosis was confirmed through histopathological analysis. Cellular DF are challenging to diagnose as they can mimic more aggressive tumours, such as malignant fibrous histiocytomas or dermatofibrosarcoma protuberans. While DF are common, their atypical variants, like cellular dermatofibroma, can complicate diagnosis and require timely diagnosis and effective management.

## Introduction

Dermatofibromas (DF) are commonly occurring cutaneous mesenchymal lesions of the dermis presenting as solitary, firm subcutaneous raised nodules measuring less than or equal to one centimetre in diameter. These benign lesions represent ~3% of skin biopsies globally. Predominantly observed in individuals aged 20–40 years, they tend to exhibit a slight to a significant female predominance [1, 2].

Cellular dermatofibroma is a morphological variant of the typical DF characterized by frequent involvement of the deeper layers of skin and a higher likelihood of local recurrence (25%) [3]. Histologically, dermatofibroma is characterized by a confined proliferation of spindle-shaped fibrous cells intermixed with histiocytoid cells within the dermis. These nodular formations typically feature moderately defined borders with a spiculated appearance and may exert a pushing effect on surrounding tissue [2]. Although generally considered benign, in rare instances, these can undergo sarcomatous transformation, leading to a more aggressive behaviour with diagnostic challenges. Here, we present a case report of an unusual variant of DF, i.e. cellular dermatofibroma.

## Case presentation

A previously healthy, 26-year-old female presented to the emergency department at our hospital with a six-month history of a foot ulcer on her left leg. This ulcer has progressively increased in size since its onset. It recently became associated with pain and limitation of movement. The development of this ulcer was preceded by left leg swelling, but no trauma or bleeding history was reported. The patient denied any constitutional manifestations such as fever, weight loss, night sweats, or chills. She has no personal history of chronic illnesses, nor any personal or family history of malignancies. She never smoked nor consumed alcohol.

**Figure 1:**
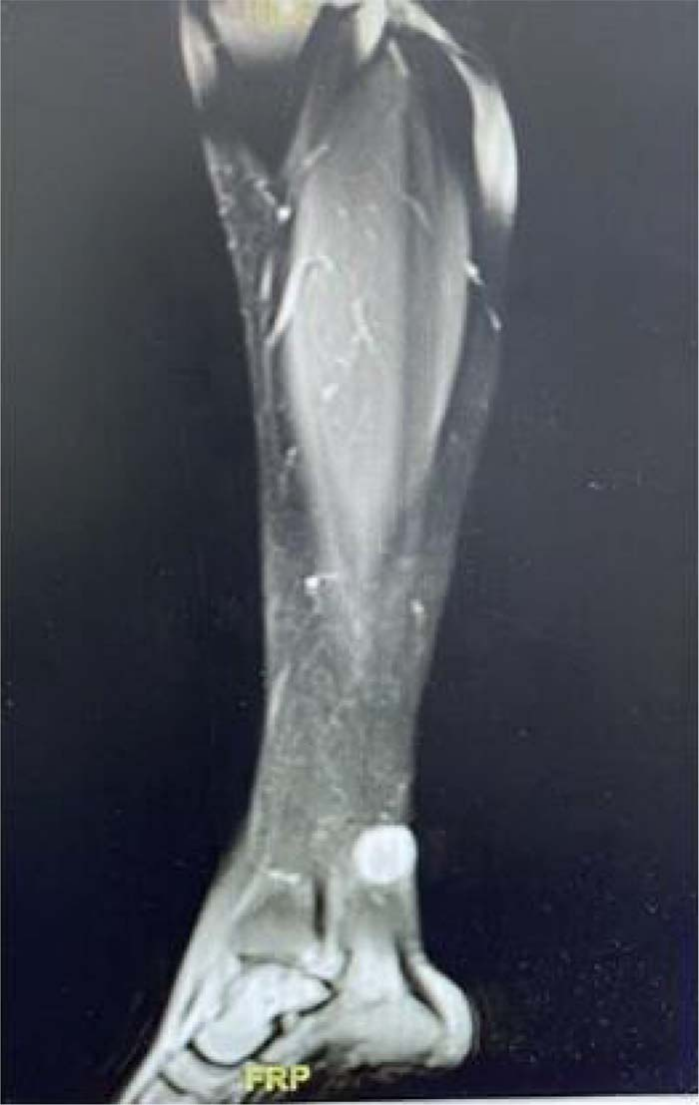
MRI demonstrating subcutaneous soft tissue mass on the posterior aspect of the lower left leg.

Upon general examination, the patient was conscious and vitally stable. Local examination of the ulcer revealed an irregular shape with black margins and brownish skin discolouration on the lower aspect of the left leg over the Achilles tendon. On palpation, tenderness, spontaneous bleeding, and pus discharge were noted. Laboratory investigations were unremarkable. A complementary magnetic resonance imaging (MRI) was performed and demonstrated a subcutaneous soft tissue mass on the posterior aspect of the lower leg, adjacent to the Achilles tendon, measuring 1.9 × 2.1 × 2 cm in dimensions as shown in Fig. 1a.

The patient underwent an excision with primary closure under general anaesthesia. Post-operative course was unremarkable, and patient was discharged home with a follow-up appointment.

**Figure 2:**
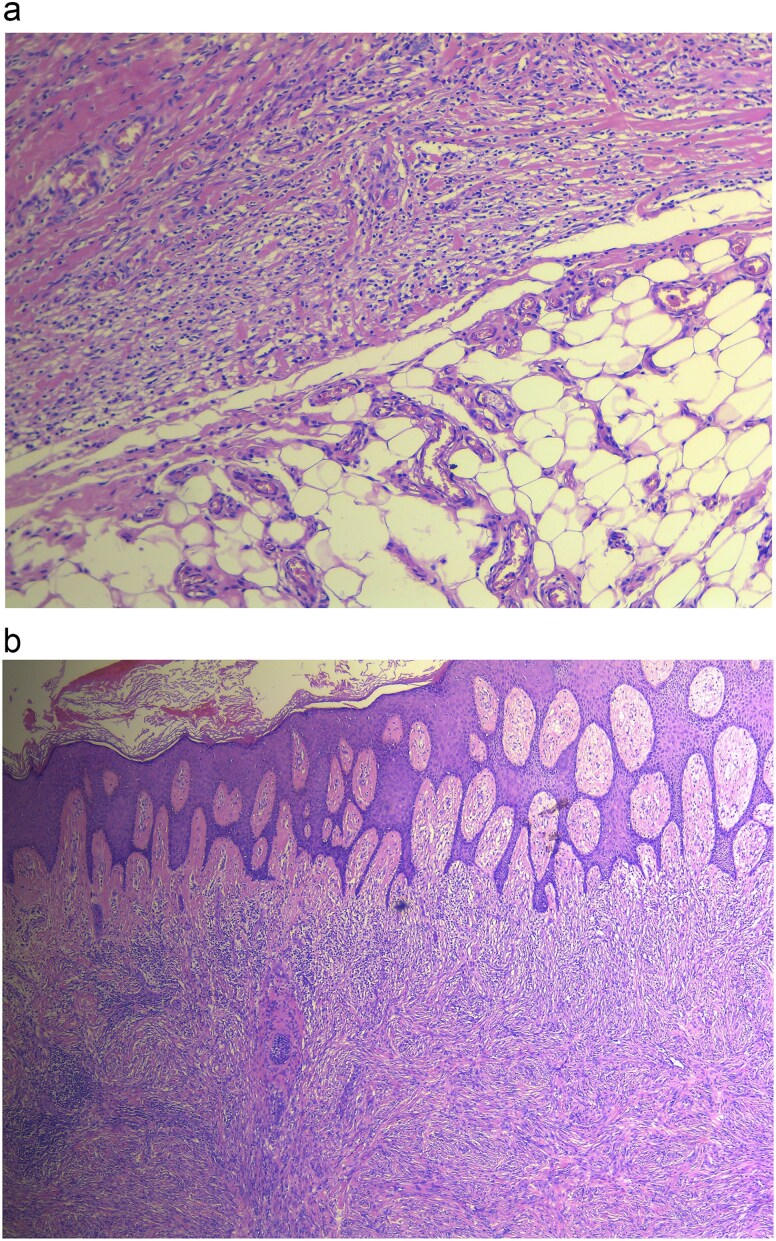
(a) Histopathology revealing cellular DF showing no infiltration, (b) DF showing dermal lesion.

Histopathology revealed gross findings of an ulcerated mass measuring 2 × 2 cm. Microscopic findings showed a well-circumscribed spindle cell neoplasm extending from a hyperplastic ulcerated epidermis into the subcutaneous tissue, with the floor of the ulcer demonstrating rich vasculature. The neoplasm is composed of packed interlacing fascicles of spindle cells with a large focus of necrosis, as seen in Fig. 2a and b. Additionally, inflammatory cell aggregates, rich in plasma cells, were found within the peripheries of the neoplasm. Features such as pleomorphism, nuclear atypia, multinucleated cells, and abnormal mitotic figures were not significantly noticeable.

Furthermore, biomarker testing of the specimen was positive for Vimentin, SMA and CD10 and negative for S100, CK516, CD31, CD34, HHV8, CK, Desmin, CD45, Melan A, BCL2, CD99, and H Caldesmon. All the previously mentioned findings were consistent with the diagnosis of cellular DF.

## Discussion

Soft tissue tumours are relatively rare and present significant challenges in accurately assessing the distribution between benign and malignant lesions. Over 50 different soft tissue tumours have been recorded in the literature, with their management depending on their histopathology and immunohistochemistry [2, 4, 5]. DF, a type of soft tissue mass found in the skin, exhibit significant histopathological diversity. Medical literature extensively documents its different variants, such as typical, aneurysmal, hemosiderotic, epithelioid, cellular, atrophic, clear cell, and lipidized fibrous histiocytoma [6, 7]. In our case, we observed a cellular dermatofibroma variant indicating an undifferentiated mesenchymal origin that differentiates along a wide spectrum encompassing fibroblastic and histiocytic features. These cellular DF exhibit characteristics that can be both benign and occasionally exhibit malignant patterns, a rarity in documented cases [7].

This unusual variant, recorded in our case, presented as a soft tissue mass of irregular shape with black margins. Features of tenderness, bleeding, and pus discharge were noticed upon palpation. Microscopic examination revealed a well-defined spindle cell neoplasm extending from a hyperplastic ulcerated epidermis into the subcutaneous tissue. The tumour consisted of densely packed interlacing fascicles of spindle cells, with a notable area of necrosis. Importantly, there were no significant features of pleomorphism, nuclear atypia, multinucleated cells, or abnormal mitotic figures, which are typically associated with malignant fibrous histiocytoma. This case also excluded the differential diagnosis of dermatofibrosarcoma protuberans with the absence of subcutaneous replacement, storiform growth pattern, and monomorphism observed in typical cases of that entity. Similarly, the typical leiomyosarcoma characteristics (such as spindle cells with cigar-shaped nuclei and cytological atypia) did not correspond to the histopathological findings in our case, thereby making it an unlikely differential [7–11].

A retrospective review conducted in 2019 by Siegel *et al.*, consisting of 93 cases of cellular DF showed similar histopathological characteristics as seen in our case. Our specimen was positive for Vimentin, SMA and CD10 and negative for S100, CK516, CD31, CD34, HHV8, CK, Desmin, CD45, Melan A, BCL2, CD99, and H Caldesmon, closely aligning with the typical profile seen in cellular DF cases, particularly with positive results for Vimentin and negative results for S100, Desmin, CD34, and CD31. These findings were consistent with those found in the aforementioned review, where CD34 negativity and S100 negativity were common [12]. In terms of treatment, the preferred approach for DF is local excision with clear surgical margins. If not completely excised, it has been reported to show a higher recurrence rate. However, there remains controversy over the optimal management of cellular DF. Some authors recommend complete excision or Mohs micrographic surgery, particularly for cases showing aggressive growth patterns [3, 13].

## Conclusion

Our case highlights the diagnostic and management challenges of unusual variants of cellular DF, which have high rates of recurrence among soft tissue tumours. Given its complexity, further research should focus on deepening understanding and thereby enhance diagnostic accuracy and refine management guidelines. To ensure optimal patient care, it is essential to implement comprehensive diagnostic protocols and conduct rigorous long-term follow-up.

## Patient's perspective

Our patient was satisfied with the treatment and attended her follow-ups regularly with no evidence of disease recurrence or metastasis postoperatively.
